# Quality and Quantity of School Lunch in Nanjing: Based on Data from the Sunshine Restaurant Supervision Platform

**DOI:** 10.3390/nu16142184

**Published:** 2024-07-09

**Authors:** Xiaofang Lin, Yuanyuan Li, Qiong Wu, Yizhou Lv, Yirong Zhu, Jingwen Liu, Le He, Zhixu Wang

**Affiliations:** 1 Department of Maternal, Child and Adolescent Health, School of Public Health, Nanjing Medical University, Nanjing 211166, China; xiaofanglin@njmu.edu.cn (X.L.); lyy272829@stu.njmu.edu.cn (Y.L.); zyr030728@njmu.edu.cn (Y.Z.); ljw@stu.njmu.edu.cn (J.L.); HELe2003@stu.njmu.edu.cn (L.H.); 2 Nanjing Municipal Healthcare Institute for Primary and Secondary Schools, Nanjing 210002, China; njwbslyz@126.com (Q.W.); 13584039900@139.com (Y.L.)

**Keywords:** students, school lunch, nutrition intake, nutritive quality

## Abstract

School lunch plays an important role in children’s healthy growth. Previous investigations revealed many problems with school lunches, including unreasonable dietary structure and insufficient micronutrients. This study aimed to assess the dietary structure and nutritional quality of lunches in Nanjing primary and middle schools. A stratified cluster random sampling method was used to select 44 schools that supply lunch in 12 districts in Nanjing, with two primary and two middle schools in each district. Twenty-four primary and twenty middle schools were selected. The Mann–Whitney U test was used to explore the influencing factors. Findings revealed a serious shortage of milk and fruit in school lunches; supply of eggs, fish, shrimp, and shellfish was less than half of the recommended quantity; livestock and poultry supply exceeded the recommended level by over four times. Energy and nutrition intake were suboptimal. Provision of energy, carbohydrates, vitamins (A, B1, B2, and C), calcium, and iron in urban primary schools was significantly higher than that in non-urban primary schools. The same pattern of significantly higher nutrients was equally seen in urban middle schools compared with non-urban middle schools, indicating that food supply was affected by regional economies. Therefore, it is urgent to improve the quality of lunches, with a particular focus on those in non-urban areas.

## 1. Introduction

Children’s access to a balanced diet is the basis for continuously improving national health and comprehensively promoting the smooth implementation of the Healthy China 2030 plan. Children are in a critical period of physical growth and intellectual development [1], with a strong metabolism and a significant increase in demand for energy and nutrients [2]. Malnutrition in childhood is associated with the development of diseases in young adulthood and an increased risk of certain diseases such as obesity, cardiovascular disease, and cancer in adulthood [3,4,5,6], which places a huge burden on the family and society. 

China is a developing country in which problems of excessive and insufficient nutrition in children coexist [7]. On the one hand, the incidence of overweight and obesity is gradually increasing. However, micronutrient deficiencies persist in children, especially in terms of vitamin A and calcium intake. According to a survey conducted between 2015 and 2019, the overweight rate of children and adolescents aged 6–17 years was 11.1%, while the prevalence of obesity was 7.9% [8]. The proportion of Chinese children with marginal vitamin A deficiency increases with age and shows distinct regional and age-related disparities. Even in large cities like Beijing, a survey among preschool children indicates a vitamin A deficiency rate of 9.84% and a marginal deficiency rate as high as 43.49% [9]. The calcium intake was generally low. The prevalence of calcium insufficiency in Chinese children exceeds >90% and increases with age [10]. 

Children spend most of their time at school [11], and lunch is their main meal during school hours and occupies an important position in their dietary intake. Many countries have developed school feeding programs [12,13,14]. The Chinese school lunch program, initiated in 1987, is a service organized by schools under the guidance of the government to improve the nutritional status of students. The main feeding methods include the independent operation of school canteens and the distribution of external food delivery enterprises, and the school is mainly self-operated. China’s school lunch program began in 1987, when the economic level was low and the popularity of the program was limited. In 2011, China’s large-scale Student Nutrition Improvement Program improved the nutritional status of primary and middle school children. By the end of 2021, the program had covered more than 40 million students, with the government investing nearly 197 billion RMB [15]. With the development of the social economy and government guidance in nutrition and hygiene, the nutritional quality of Chinese school lunches has been continuously improving. However, unbalanced nutrition in lunch remains a problem. As shown by research on the nutritional quality of school lunches, school lunches in urban and non-urban areas generally have an insufficient supply of aquatic products, vegetables, beans, milk, and other foods [16,17,18,19]. Lunch provides 35–40% of an individual’s daily energy and various nutrients, and its nutritional quality is crucial for children’s growth and development. Han Liu et al. [20] showed that the intake of energy, vitamin A, vitamin C, and iron in school lunches correlated with the height and weight of students. Schools are ideal natural environments for public health interventions [21]. Intervention studies on school lunches have shown that improving the quality of school lunches not only improves the intake of related nutrients but also promotes student growth and development [22,23]. By collecting the dietary information of 44 primary and secondary schools in Nanjing, this study aimed to understand the characteristics of the school lunch dietary structure to improve the quality of school lunches and meet the needs of children’s growth and development. 

## 2. Materials and Methods

### 2.1. Sample Collection

Stratified cluster random sampling was used to select two middle schools and two primary schools from each district in Nanjing. A total of 48 schools were selected as survey sites, of which one non-urban primary school and three non-urban middle schools were excluded for not providing the required food consumption data in a timely manner as stipulated during the survey period. Finally, a total of 24 primary schools and 20 secondary schools (one district had three primary schools and one middle school) were selected, with a total of 64,933 students participating in the school lunch program (Table 1).

### 2.2. Data Collection and Evaluation Methods

Using the Information Supervision Platform of Jiangsu Primary and Middle School Sunshine Canteens, we recorded the weight of all ingredients purchased by each school every week, based on which we calculated the total amount of food consumed by the school every week. By combining the number of students eating and the number of meals served per week, we calculated the actual food supply for each student per meal. To exclude the interference of seasonal factors, we extracted data recorded in the second week of the month (March, June, September, and December) using the platform to calculate the actual food supply per student per meal in 44 schools. This study was approved by the Nanjing Medical University Ethics Committee.

Based on the WS/T 554-2017 Nutrition Guide for Student Meals [24], the recommended daily allowance for each type of food was calculated, and the energy and nutrient allowance for all foods was calculated according to the China Food Composition Table (6th Edition) [25]. The recommended energy and nutrient values were calculated according to the DIETRY REFERENCE INTAKES FOR CHINA (DRIs) (2023 edition) [26], as lunch accounts for 40% of the total daily supply. The reference value is the interval range, which is taken as half the sum of the maximum and minimum values. The reference ranges for food types, energy, and nutrients for primary school students were 6–11 years old, and for middle school students were 12–14 years old. The energy supply was based on the reference value for boys. The evaluation criteria for food suitability were as follows: a food supply with a recommended value between 80% and 120% was considered adequate, while a value below 80% was considered inadequate, and a value above 120% was considered excessive. Evaluation criteria for the suitability of energy and nutrient supply were as follows: energy supply reaching 90–110% of the recommended value is adequate, below 90% is inadequate, and above 110% is oversupply. Protein supply should reach 80–120% of the recommended value for adequacy; below 80% is inadequate, and above 120% is oversupply; 60% of the recommended value of other nutrients is adequate, and below 60% is inadequate [16]. 

The suitability of the food type, energy, and nutrients was expressed as a percentage of the recommended value. Compliance rates of food types and energy and nutrient supply were expressed as the number of schools meeting the recommended standards as a percentage of the total number of schools [Compliance rate of food or energy and nutrient supply = (number of schools within the appropriate range of food or energy and nutrient supply/total number of schools) × 100%]. 

### 2.3. Statistical Analysis

Statistical analyses were performed using SPSS (version 27.0) (IBM Corporation, Armonk, NY, USA). The data did not follow a normal distribution when analyzed using the Shapiro–Wilk test. Thus, the median was used to represent the data for each food type and energy source. The Mann–Whitney U test was used to compare differences among the different regions. The Kruskal–Wallis H test was used to compare the differences between quarters, and Bonferroni correction was performed on the results. Statistical significance was set at *p* < 0.05. 

## 3. Results

### 3.1. Food Supply

The survey found that in four quarters of 44 primary and middle schools, 68% did not supply fruit in the first quarter, 72.9% did not supply eggs in the second quarter, more than 59% did not supply milk in all seasons, and 100% exceeded the recommended upper limit for livestock and poultry supply in all seasons.

**Figure 1:**
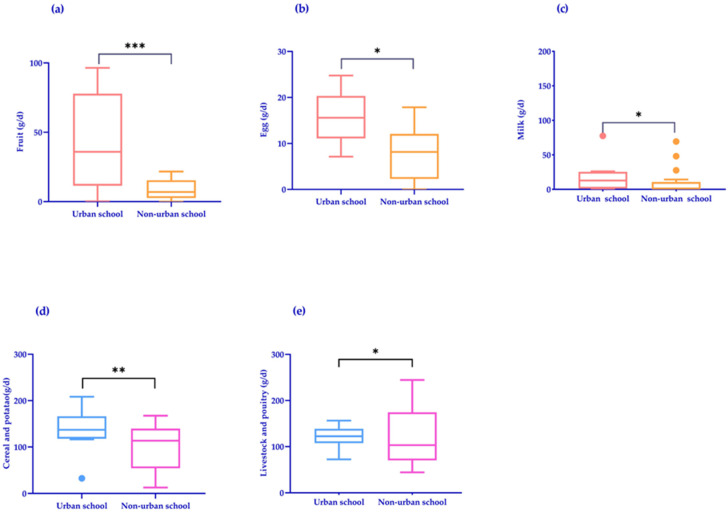
Comparison of food supply in different regions. (**a**–**c**) Comparison of urban and non-urban parts in primary schools. (**a**) Fruit; (**b**) egg; and (**c**) milk. (**d**,**e**) Comparison of urban and non-urban parts in middle schools. (**d**) Cereal and potato. (**e**) Livestock and poultry. Outliers are plotted as dots. *p* < 0.05 indicates significance; * *p* < 0.05; ** *p* < 0.01; and *** *p* < 0.001.

The median coarse grain supply in primary schools was 4.4 g and potatoes 8.2 g, while the median coarse grain supply in middle schools was 2.5 g and potatoes 15.7 g. Given the coarse grain supply was extremely low, coarse grains were combined into cereal and potatoes (cereal and potato in the following article included cereals, potatoes, and coarse grains). The supply of cereals and potatoes, fruits, fish, shrimp, shellfish, eggs, soy and nuts, and milk failed to reach the recommended value; the supply of fruit was less than 20% of the recommended value, the milk supply was almost zero, and the supply of livestock and poultry food was more than four times the recommended value. Compared with non-urban primary schools, the supply of fruits, eggs, and milk in urban primary schools was significantly higher than that in non-urban primary schools (*p* < 0.05). Also, the supply of cereals and potatoes and livestock and poultry food in urban middle schools was significantly higher than that in non-urban middle schools (*p* < 0.05) (Table 2) (Figure 1). 

When comparing the food supply of different regions in the same quarter, it was found that the fruit supply in urban primary schools was higher than that in non-urban schools in all quarters. Milk supply was seasonal, while primary milk supply in non-urban primary schools was lower in all quarters. When comparing middle schools, cereal and potato supplies in urban secondary schools were significantly higher than those in non-urban schools in the second quarter (*p* = 0.007) (Table S1).

**Figure 2:**
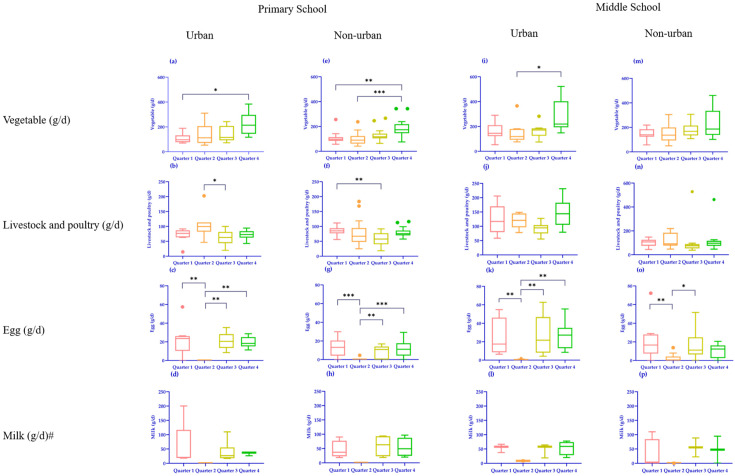
Comparison of food supplies in different seasons in the same area. (**a**–**d**) Comparison of food supply in different seasons in urban primary schools. (**e**–**h**) Comparison of food supply in different quarters in non-urban primary schools. (**i**–**l**) Comparison of food supply in different quarters in urban middle schools. (**m**–**p**) Comparison of food supply in different quarters in non-urban middle schools. # Considering only the number of schools with milk supply, the sample sizes of urban primary schools supplying milk in four quarters were 5, 0, 6, and 3, and non-urban primary schools were 4, 0, 4, and 4, respectively. The sample sizes of urban middle schools were 3, 1, 3, and 4, while non-urban middle schools were 4, 1, 2, and 2. Outliers are plotted as dots. *p* < 0.05 indicates significance; * *p* < 0.05; ** *p* < 0.01; and *** *p* < 0.001.

A statistically significant difference was observed in vegetable supply between the first and fourth quarters of urban primary schools (*p* = 0.028) when comparing different quarters in the same area. The egg supply in the second quarter was significantly different from that in the other three quarters, and the milk supply in the second and third quarters was also significantly different (*p* = 0.034). In non-urban primary schools, vegetable consumption was significantly higher in the fourth quarter than in the first and second quarters; fruit, livestock, and poultry meat supplies were higher in the first quarter than in the third quarter. The inter-quarter difference in egg supplies in non-urban primary schools was consistent with that in urban primary schools, and the second quarter was significantly lower than in other quarters. The supply of vegetables in the second quarter was significantly lower than that in the fourth quarter (*p* = 0.022), and the supply of eggs in the second quarter was significantly lower than that in the other quarters. Egg availability in non-urban secondary schools was significantly lower in the second quarter than in the first or third quarters (Table S1) (Figure 2).

### 3.2. Compliance Rate of Food Supply

**Figure 3:**
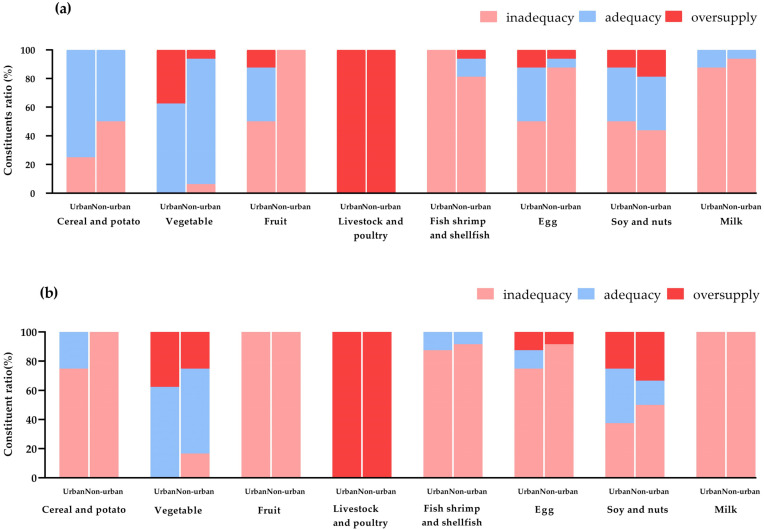
Food supply in primary and middle schools in different regions. (**a**) Primary schools. (**b**) Middle schools. Unit of measurement—(g).

Fruit, fish, shrimp, shellfish, eggs, and milk were in short supply in more than 70% of the primary schools. All schools had an oversupply of livestock and poultry of up to 482.9% of the recommended value. In over 80% of the middle schools, fruit, fish, shrimp, shellfish, eggs, and milk were in short supply, whereas the livestock and poultry supply significantly exceeded the recommended upper limits. The number of schools that met the recommendations for fruit supply in urban primary schools was significantly higher than that in non-urban primary schools (*p* = 0.008). In contrast, there was no significant difference in the food compliance rate in middle schools (Figure 3).

### 3.3. Energy and Nutrient Availability

Table 3 shows that the energy supply in urban schools was significantly higher than that of non-urban schools (*p* < 0.05). The protein and fat supply in primary and middle schools was higher than the recommended standards, and the energy supply ratios of protein, fat, and carbohydrates did not meet the recommended standards. In terms of micronutrients, the calcium supply was low in both primary and middle schools. Vitamin A levels were inadequate in the middle and non-urban primary schools. The levels of energy, carbohydrates, vitamins (A, B_1_, B_2_, and C), calcium, and iron in urban primary schools were higher than those in non-urban primary schools (*p* < 0.05). Similarly, the levels of energy, protein, carbohydrates, vitamins (B_1_, B_2_), iron, and zinc were higher in urban middle schools than in non-urban middle schools (*p* < 0.05). 

When comparing the same quarters in different regions, it was found that urban primary schools had a significantly higher energy supply in the second quarter, vitamin A supply in the first quarter, and vitamin C supply in the second and fourth quarters than non-urban primary schools (*p* = 0.045, *p* = 0.007, and *p* = 0.023, respectively). The carbohydrate energy supply in urban middle schools was significantly better than that in non-urban schools in the second and third quarters, and the supply of vitamins B_1_ and B_2_ in the fourth quarter was higher than that in non-urban middle schools (*p* = 0.025, *p* = 0.047) (Table S2).

When comparing different areas in the same region, the supply of vitamins A and B_2_ in the fourth quarter of non-urban primary schools was significantly higher than that in the first and second quarters. In comparison, the supply of vitamin C in the second quarter was significantly lower than that in the third and fourth quarters. Calcium availability was significantly lower in the second quarter than in the first or fourth quarters. The iron supply in the second quarter was lower than that in the fourth quarter (*p* = 0.024); however, there was no difference in the supply of nutrients in urban primary schools. The carbohydrate-energy ratio of urban middle schools in the second quarter was significantly higher than that in the other quarters. There were statistical differences in the supply of vitamin B_1_ between the second and fourth quarters and vitamin B_2_ between the first and fourth quarters; however, there was no statistical difference in the supply of energy nutrients in non-urban middle schools (Table S2).

### 3.4. Energy and Nutrients Supply and Account for Recommended Standards

**Figure 4:**
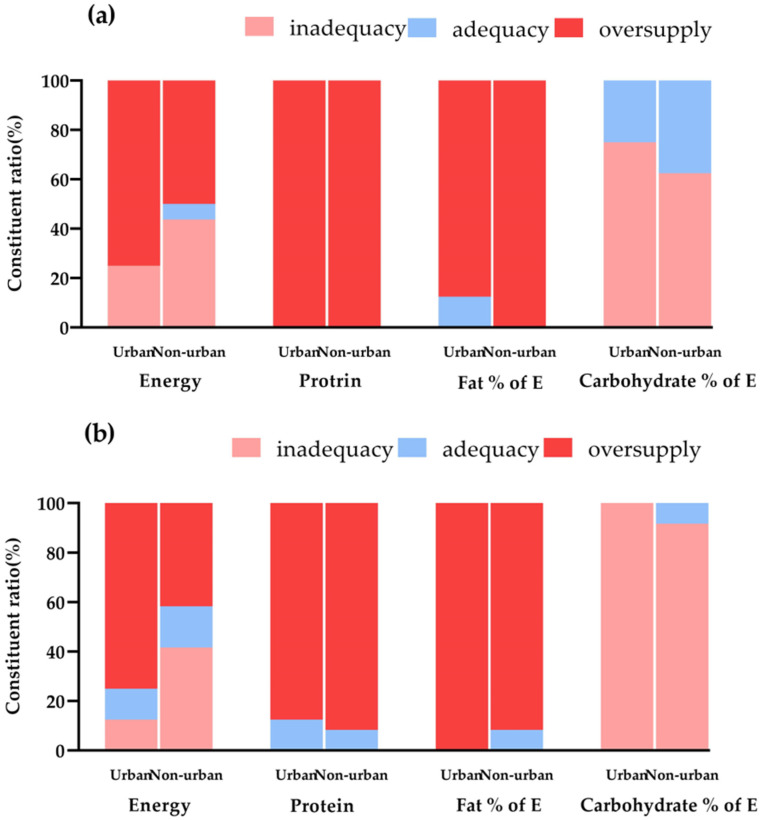
Energy supply in primary and middle schools in different regions. (**a**) Primary schools. (**b**) Middle schools.

**Figure 5:**
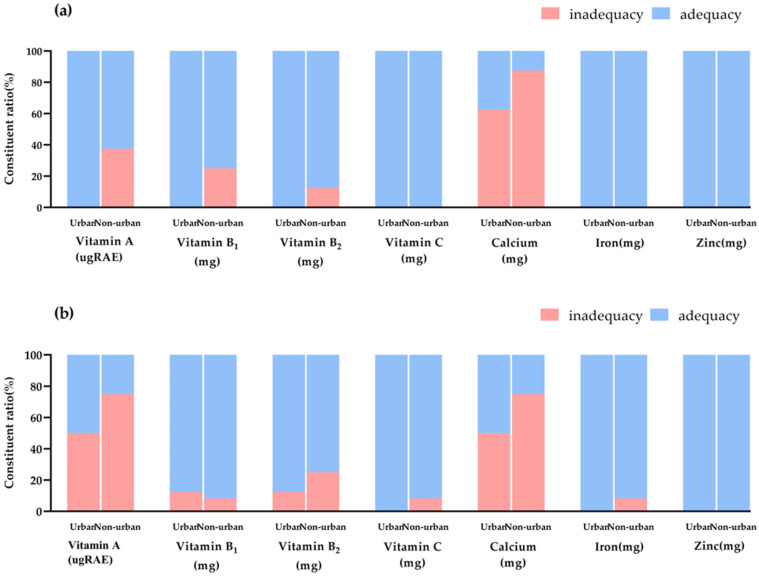
Nutrient supply in primary and middle schools in different regions. (**a**) Primary schools. (**b**) Middle schools.

More than 50% of primary and middle schools had an excessive energy supply; the energy ratio of energy-producing nutrients, such as protein and fat, was above the recommended upper limit, and more than 90% of schools had an oversupply of protein and fat. However, the amount of energy obtained from carbohydrates was lower than the recommended amount (Figure 4). Vitamin A supply was inadequate in one-third of non-urban primary schools and more than 60% of middle schools. Insufficient calcium supply was a common phenomenon in primary and middle schools, with more than 70% of the schools having an insufficient calcium supply. Among them, seven out of eight of the non-urban primary schools had a calcium supply below the recommended lower limit (Figure 5).

## 4. Discussion

The results indicate a serious shortage in the provision of milk and fruit for school lunches, alongside an inadequate supply of fish, shrimp, and shellfish, while there was a high supply of livestock and poultry. Moreover, the intake of energy and nutrients falls short of ideal levels. 

Compared to urban schools, we found that non-urban schools are more susceptible to the effects of different seasons in terms of food and nutrient supply, with the economy and season having a greater influence on school feeding. In summary, the structure of school meals remains unreasonable; therefore, it is urgent to adjust the structure of school lunches and improve their quality.

### 4.1. Serious Insufficient Supply of Milk and Fruits

Dairy products are the main dietary sources of important nutrients such as calcium, potassium, and vitamin D and are good suppliers of high-quality protein [27,28], which plays an important role in promoting the healthy growth of children and adolescents. At present, it is recommended that primary school students consume 80 g/day of milk and dairy products for lunch, while middle school students consume 100 g/day of milk and dairy products. According to the survey results, 95% of schools failed to meet the recommended value for milk and dairy products, and 59% did not provide milk and dairy products at all. At present, insufficient milk supply is a common problem in school feeding, and other related studies have reached similar conclusions [2,12,19,20,29]. The possible reasons for milk availability in schools include physiological and food safety concerns among students. Lactose is a unique carbohydrate found in human and mammalian milk. After lactose consumption, lactase is required to break down and absorb it [30]. If lactose is not completely broken down and absorbed, lactose intolerance develops. The prevalence of lactose intolerance increases with age, and the proportion of Asians exceeds 90% [31,32]. Once lactose intolerance occurs in schools, it can easily cause panic among parents and increase pressure on school principals.

Regarding food safety concerns, the milk supplied by schools in China is mainly fresh and pasteurized. The price of fresh milk is preferential; however, the storage and transportation conditions are high, and the storage time is short. When the temperature during storage fails to meet these requirements, food can spoil and deteriorate within a short time [33], causing food safety problems. In this case, the school avoided supplying milk. However, previous studies have shown that the overall milk intake of Chinese children is insufficient [34,35]. Milk has a high nutritional value. Long-term lack of milk cannot meet children’s growth and development needs, which may affect their growth and development. Therefore, there is an urgent need to introduce suitable dairy products into school lunches.

Cheese is a nutritious and well-tolerated fermented dairy product formed from the fermentation of milk [36], especially proteins and calcium. Due to its high digestibility, it is an ideal source of protein and calcium. In addition, after whey and long-term fermentation, most of the lactose is excreted or converted to lactic acid; the content is low (1–3%), not easy to sensitize, and suitable for consumption by lactose-intolerant people. Schools can supply cheese instead of liquid milk to ensure sufficient nutrition and overcome the difficulty of providing liquid milk.

The survey found that only two primary schools reached the recommended fruit supply level. Fruit is rich in vitamins, minerals, dietary fiber, and other antioxidant substances that promote appetite and digestion. Some studies have shown that fruits are important for maintaining bone health [37]. However, the fruit has disadvantages such as difficult preservation, transportation, and high costs. Schools can provide fruits such as apples, oranges, and bananas that are affordable, easy to transport, and preserved. If schools are unable to provide fruit to students, parents should be educated about fruit nutrition and encouraged to provide fruit at home or bring fruit to school. This is to ensure that primary school children can obtain 80–100 g of fruit per day, and secondary school children can obtain 100–140 g of fruit per day so that children can receive adequate nutrition.

### 4.2. Imbalance in Supply of Livestock and Poultry, and Fish, Shrimps, and Shellfish

Fish, poultry, eggs, and lean meat contain high-quality protein and a variety of micronutrients that are beneficial to humans, with fish and poultry being the best and livestock being high in fat; therefore, excessive intake is not recommended. With the improvement in the economic level, refined staple foods have increased in children and adolescents, and the intake of livestock and poultry meat has increased [38]. In this survey, the supply of livestock and poultry food in both middle and primary schools reached four times or more than the recommended value. In terms of nutrients, fat intake exceeded the upper limit of the highest recommended value.

However, the supply of fish, shrimp, and shellfish was less than half of the recommended value. The low supply of fish, shrimp, and shellfish may have been related to their physiological characteristics. Fish and shrimp contain bones and shells, which pose great safety hazards for students. Younger student groups were unable to spit fish and peel shrimp shells. In this situation, schools can provide parents with education on fish, shrimp, and shellfish nutrition; promote the supply of fish, shrimp, shellfish, and other food in the family; and, most importantly, strengthen education on related food for students and improve their eating skills. Schools can also purchase processed fish, shrimp, and shellfish, such as fish steaks with fish bones removed and shrimp and shellfish with shells removed; however, they should be careful to prevent food safety problems. They should pay attention to skills in the cooking process and ensure food safety while increasing the supply so that the weekly supply meets an average of 16–20 g of fish, shrimp, and shellfish per day.

### 4.3. Insufficient Supply of Cereals and Potatoes

The supply of cereals and potatoes in primary and middle schools was lower than the recommended value. In particular, the supply of cereals and potatoes in non-urban middle schools was less than 60% of the recommended value. Significant differences were observed among the economic regions. In this study, the staple foods of primary and middle schools were mainly cereals, and the supply of coarse grains was extremely low, which is similar to previous research results [39,40]. Compared to rice, noodles, and other fine foods, coarse grains have a higher dietary fiber content and lower glycemic index, which are conducive to the control of obesity, type 2 diabetes, and cardiovascular diseases [41,42]. The appropriate use of cereals and potatoes instead of rice as staple foods is conducive to the healthy growth of children and adolescents.

### 4.4. Inadequate Supply of Calcium and Vitamin A

Calcium is involved in physiological activities such as nerve conduction and muscle contraction in the human body and regulates cell viscosity, blood coagulation, and structural support of bone composition [43]. Food is the preferred source of calcium, and milk is the best food source. In this survey, the calcium supply was less than 60% of the recommended value because the milk supply was extremely low. According to a study by the United States Department of Agriculture, the calcium content of 100 g of milk can reach 100 mg, and a cup of 300 mL of milk can meet 30% of the calcium requirements for children and adolescents. Other foods, such as green leafy vegetables, beans, and cereals, can provide calcium, but the calcium content per serving is usually lower than that of dairy products [44]. It is necessary to provide students with a balanced and nutritious diet, especially dairy products, to improve school lunches and promote the healthy growth of students.

Vitamin A, also known as retinol, is an essential fat-soluble vitamin for the human body; deficiency can cause dry eye syndrome and night blindness [45] and even affect children’s cognitive function and developmental level. However, vitamin A cannot be synthesized in the human body, and dietary intake is required to meet the body’s needs. Foods rich in vitamin A are mainly animal foods such as liver, cod liver oil, dairy products, and eggs, whereas plant foods can only provide carotenoids [46]. Visceral foods were almost unavailable in primary and junior high schools; therefore, such foods were included in the livestock and poultry meat to calculate nutrients. More than 80% of schools provided a lower vitamin A supply than recommended, which may be related to an insufficient supply of liver and eggs in primary and secondary schools. It is recommended that schools add foods rich in vitamin A, such as pig liver or chicken liver, to lunch once a week.

### 4.5. Strengths and Limitations

To ensure the data were representative, we used stratified cluster random sampling to select the surveyed schools. To control the error caused by quarterly reasons, we selected the data of the second week of March, June, September, and December among the four quarters for statistical analysis. This study has some limitations. First, the data came from the information supervision platform of the canteen, thereby introducing certain limitations, i.e., our analysis is based on the weight of input and output, and the intake of energy and nutrients is calculated under the assumption that students have no leftovers, so it can only be used as a basis for assessing the dietary structure and cannot represent the actual intake level of students. However, when students’ food intake was overestimated, the intake of milk, fruit, fish, shrimp, and shellfish was seriously deficient, and many nutrients remained insufficient, indicating that the dietary structure of school lunches was unreasonable and needed improvement. Second, the survey was conducted in Nanjing; therefore, its generalizability to other cities is limited. However, some research results, such as an excessive supply of livestock and poultry and a serious shortage of fruit and milk, are similar to those of other areas, indicating that the problem of an unreasonable dietary structure is common.

## 5. Conclusions

In conclusion, there are some problems with the lunches provided by primary and middle schools in Nanjing. For example, the supply of fruit, fish, shrimp, shellfish, and milk is severely inadequate, whereas that of livestock and poultry meat significantly exceeds the recommended standards. However, the supply of vitamin A, calcium, and other nutrients was insufficient. We attribute these variances to three principal factors. Initially, schools prioritize safety above nutritional equilibrium, potentially resulting in a conservative food selection strategy that emphasizes risk minimization over nutritional optimization. Additionally, a common misconception among parents about the nutritional value of meat has resulted in an oversupply of poultry and livestock products in schools. Finally, the dearth of qualified nutritionists in schools leads to an absence of expert guidance for crafting well-rounded meal plans. Schools and relevant departments should focus on these issues. Education and health departments should strengthen supervision over the quality and quantity of school lunches, strengthen the collaboration of family and school to ensure that students receive the energy and nutrients needed for growth and development, and promote the healthy growth of students. Later research could be undertaken in the following areas. Firstly, intervention studies to evaluate the effectiveness of nutrition education programs or policy changes aimed at improving the quality of school meals. Furthermore, comparative studies between different regions or countries and China to assess variations in school lunch programs and their impact on children’s nutrition. Qualitative research should also be conducted to explore the perceptions and preferences of students, parents, and school staff regarding school lunches.

## Figures and Tables

**Table 1:** Characteristics of schools and students.

	Total		Urban		Non-Urban	
	Number of Schools	Number of Students	Number of Schools	Number of Students	Number of Schools	Number of Students
Primary school	24	37,931	8	12,765	16	25,166
Middle school	20	27,002	8	10,057	12	16,945

**Table 2:** Actual food supply and percentage of recommended value in schools in different districts (%).

Category	Primary School (n = 24)					Middle School (n = 20)				
	Recommended	Urban	Non-Urban	Z	*p*- Value	Recommended	Urban	Non-Urban	Z	*p*- Value
Cereal and potato	120	89.9 (74.9)	73.8 (61.5)	0.979	0.327	150	138.9 (92.6)	82.2 (54.8)	3.138	**0.002**
Vegetable	140	120.8 (86.3)	115.2 (82.3)	0.801	0.423	170	173.2 (101.9)	154.9 (91.1)	0.314	0.753
Fruit	80	35.2 (44.0)	3.2 (4.0)	5.055	**<0.001**	110	3.4 (3.1)	2.5 (2.3)	0.385	0.701
Livestock and poultry	16	77.3 (482.9)	76.2 (476.3)	0.451	0.652	22	112.2 (510.0)	91.5 (451.9)	2.347	**0.019**
Fish, shrimp, and shellfish	16	6.3 (39.4)	4.5 (28.1)	0.846	0.398	22	8.0 (36.4)	4.0 (18.2)	1.892	0.059
Egg	20	15.3 (76.5)	6.2 (31.0)	2.530	**0.011**	30	13.5 (45.1)	10.6 (35.3)	1.212	0.226
Soy and nuts	13	7.9 (60.8)	10.1 (77.7)	1.438	0.150	16	11.5 (71.9)	13.2 (82.5)	0.629	0530
Milk	80	0.0 (0.0)	0.0 (0.0)	2.235	**0.025**	100	0.0 (0.0)	0.0 (0.0)	1.724	0.085

Data are presented as medians (%). n—number of schools. Bold font indicates statistically significant differences. Unit of measurement—(g). The percentage of recommended value is calculated as follows: (actual supply/recommended standard value) × 100%.

**Table 3:** Supply of energy and nutrients and compared to DRIs-2023 (%).

Item	Primary School (n = 24)					Middle School (n = 20)				
	DRIs	Urban	Non-Urban	Z	*p*-Value	DRIs	Urban	Non-Urban	Z	*p*-Value
Energy (kcal)	660	807.1 (122.3)	666.7 (101.0)	2.440	**0.015**	920	1115.9 (121.3)	768.1 (83.5)	2.907	**0.004**
Protein (g)	15	32.1 (214.1)	30.5 (203.3)	0.987	0.324	22	48.0 (218.2)	33.9 (154.1)	2.347	**0.019**
Protein % of E	-	16.3	18.4	2.588	**0.010**	-	17.1	16.8	0.697	0.200
Fat % of E	20–30	35.7	38.8	1.135	0.256	20–30	38.6	40.8	1.237	0.186
Carbohydrate (g)	48	98.2 (204.6)	77.2 (160.8)	2.386	**0.017**	60	140.4 (233.9)	86.5 (144.2)	3.428	**<0.001**
Carbohydrate % of E	50–65	48.8	43.1	1.710	0.087	50–65	45.6	40.0	1.974	**0.031**
Vitamin A (ug RAE)	198	188.5 (95.2)	106.0 (53.5)	3.163	**0.002**	312	153.2 (49.1)	148.9 (47.7)	1.257	0.209
Vitamin B_1_ (mg)	0.42	0.45 (107.1)	0.37 (88.1)	2.526	**0.012**	0.56	0.64 (114.3)	0.49 (92.5)	2.583	**0.010**
Vitamin B_2_ (mg)	0.42	0.42 (100.0)	0.31 (73.8)	2.573	**0.010**	0.56	0.56 (100.0)	0.36 (64.3)	2.416	**0.016**
Vitamin C (mg)	28	51.3 (183.3)	38.9 (139.0)	3.000	**0.003**	38	47.6 (125.1)	48.2 (126.9)	0.000	1.000
Calcium (mg)	360	188.7 (52.4)	150.3 (41.8)	2.573	**0.010**	400	215.4 (53.9)	188.5 (47.1)	1.424	0.154
Iron (mg)	5.6	7.7 (138.2)	6.7 (119.6)	2.738	**0.017**	7.2	10.0 (139.2)	8.3 (115.3)	2.092	**0.036**
Zinc (mg)	2.8	4.8 (170.0)	4.1 (146.4)	1.593	0.111	3.4	7.6 (222.4)	5.3 (155.9)	2.789	**0.005**

Data are presented as median (%). *p*-value < 0.05 indicates significance; n—number of schools; RAE—retinol activity equivalent. Bold values indicate statistically significant differences. Unit of measurement—(g). The percentage of recommended value is calculated as follows: (actual supply/recommended standard value) × 100%. E—energy; % of E—the contributes to the percentage of energy.

## Data Availability

The data presented in this study are available upon request from the corresponding author due to (the data are not publicly available).

## Supplementary Materials

The following supporting information can be downloaded at: https://www.mdpi.com/article/10.3390/nu16142184/s1. Table S1: comparison of food types between different districts and seasons; Table S2: Seasonal comparison of energy and nutrients in different primary and middle school districts and their recommended values (%).
