# Supplementary material for: Quality and Quantity of School Lunch in Nanjing: Based on Data from the Sunshine Restaurant Supervision Platform
Source: Nutrients. 2024 Jul 9;16(14):2184. doi: 10.3390/nu16142184 (PMC11280376; doi:10.3390/nu16142184)
Supplement: Supplementary file 1 [file nutrients-16-02184-s001.zip › nutrients-3070845-supplementary.pdf]

Supplementary Tables

Table S1. Comparison of food types between different districts and seasons

	Primary School (n = 24)								Middle School (n = 20)							
	Quarter 1		Quarter 2		Quarter 3		Quarter 4		Quarter 1		Quarter 2		Quarter 3		Quarter 4	
Category	Urban	Non-urban	Urban	Non-urban	Urban	Non-urban	Urban	Non-urban	Urban	Non-urban	Urban	Non-urban	Urban	Non-urban	Urban	Non-urban
Cereal and potato	108.0 (90.0)	86.0 (71.7)	91.1 (75.9)	72.0 (60.0)	97.6 (81.3)	82.1 (68.4)	60.1 (50.1)	67.8 (56.5)	141.0 (94.0)	112.6 (75.1)	133.6 (89.1)[a]	74.5 (49.7)	143.3 (95.5)	60.4 (40.3)	134.8 (89.9)	81.5 (47.9)
Vegetable	96.4 (68.9)[b]	97.7 (69.8)[c1]	111.9 (79.9)	90.8 (64.9)[c2]	115.0 (82.1)	122.4 (87.4)	213.5 (152.5)[b]	178.6 (127.6)[c12]	144.9 (85.2)	138.9 (81.7)	118.3 (69.6)[b]	136.6 (80.4)	171.2 (100.7)	167.5 (98.5)	219.0 (128.8)[b]	186.2 (109.5)
Fruit	51.4 (64.3)[a]	0.00 (0.0)[c]	36.7 (45.9)[a]	7.0 (8.8)	27.5 (34.4)	9.3 (11.6)[c]	31.3 (39.1)[a]	0.0 (0.0)	0.0 (0.0)	0.0 (0.0)	8.8 (8.0)	2.8 (2.5)	4.1 (3.7)	6.6 (6.0)	2.2 (2.0)	3.1 (2.8)
Livestock and poultry	76.6 (478.8)	85.8 (536.3)[c]	105.5 (659.4)[ab]	66.0 (412.5)	62.8 (392.5) [b]	54.5 (340.6)[c]	72.6 (453.8)	75.7 (473.1)	117.1 (532.3)	107.6 (489.1)	120.8 (549.1)	91.9 (417.7)	94.4 (429.1)	75.7 (344.1)	143.8 (653.6)[a]	96.6 (439.16)
Fish, shrimp and shellfish	9.4 (58.8)[b]	1.6 (10.0)	0.3 (1.9)	8.0 (50.0)	6.9 (43.1)	2.7 (16.9)	6.2 (38.8)	6.7 (41.9)	7.9 (35.9)	1.8 (8.2)	7.7 (35.0)	6.8 (30.9)	13.5 (61.4)[a]	3.1 (14.1)	6.6 (30.0)	5.6 (25.5)
Egg	23.5 (117.5)[b1]	12.1 (60.5)[c1]	0.0 (0.0)[b123]	0.0 (0.0)[c123]	20.5 (102.5)[ab2]	8.4 (42.0)[c2]	18.3 (91.5)[ab3]	10.9 (54.5)[c3]	17.5 (58.3)[b1]	16.6 (55.3)[c1]	0.0 (0.0)[b12 3]	0.4 (1.3)[c12]	21.6 (72.0)[b2]	11.1 (37.0)[c2]	27.1 (90.3)[b3]	12.3 (41.0)
Soy and nuts	7.4 (56.9)	9.1 (70.0)	7.3 (56.2)	11.3 (86.9)	5.1 (39.2)	10.6 (81.5)	13.7 (105.4)	10.1 (77.7)	11.1 (69.4)	10.6 (66.3)	14.0 (87.5)	12.6 (78.8)	8.6 (53.8)	14.7 (91.9)	19.9 (124.4)	17.3 (108.1)
Milk	18.4 (23.0)	0.0 (0.0)	0.0 (0.0)[b]	0.0 (0.0)	18.4 (23.0)[b]	0.0 (0.0)	0.0 (0.0)	0.0 (0.0)	0.0 (0.0)	0.0 (0.0)	0.0 (0.0)	0.0 (0.0)	0.0 (0.0)	0.0 (0.0)	10.1 (10.1)	0.0 (0.0)

Data are presented as median (%). *p*- value <0.05 is indicative of significance; n - number of schools; (a) there are statistical differences between the same quarters in different regions; (b) there are statistical differences between quarters in urban schools; (c) there are statistical differences between quarters in non-urban schools. Unit of measurement - (g). The percentage of recommended value = (actual supply/recommended standard value) × 100%.

Table S2. Seasonal comparison of energy and nutrients in different primary and middle school districts and their recommended values (%)

Item	Primary School (n=24)								Middle School(n=20)							
	Quarter 1		Quarter 2		Quarter 3		Quarter 4		Quarter 1		Quarter 2		Quarter 3		Quarter 4	
	Urban	Non-urban	Urban	Non-urban	Urban	Non-urban	Urban	Non-urban	Urban	Non-urban	Urban	Non-urban	Urban	Non-urban	Urban	Non-urban
Energy (kcal)	746.8 (114.7)	673.2 (102.0)	860.1 (130.3)^{a}	597.9 (87.9)	787.3 (119.3)	569.3 (86.3)	808.0 (122.4)	760.9 (115.3)	995.8 (108.2)	760.7 (82.7)	1129.13 (122.7)	752.0 (81.7)	1012.9 (110.1)	698.2 (75.9)	1502.0 (163.3)	851.1 (92.5)
Protein (g)	35.9 (239.6)	32.6 (217.3)	30.7 (204.8)	26.3 (175.3)	32.0 (213.3)	25.0 (166.7)	33.7 (224.3)	31.5 (210.0)	46.9 (213.2)	40.8 (185.5)	48.1 (218.4)	37.7 (171.4)	44.6 (202.5)	29.5 (134.1)	71.7 (326.0)	38.7 （175.9)
Protein % of E	16.0	19.4	16.5	17.8	16.6	17.7	15.8	19.2	17.0	17.4	16.0	19.6	16.9	19.3	19.4	14.6
Fat % of E	35.7	36.0	24.9	33.5	41.6	43.1	39.3	47.3	41.0	40.0	27.1^{b}	34.6	38.2	45.4	42.8^{b}	43.1
Carbohydrate (g)	92.2 (192.1)	78.2 (162.9)	108.2 (225.3)^{a}	78.1 (162.7)	84.9 (176.8)	67.6 (159.3)	102.2 (212.9)	82.7 (172.3)	109.5 (182.5)	83.1 (138.5)	167.8 (279.6)^{a}	84.3 (140.5)	115.0 (191.7)^{a}	51.4 (85.7)	167.9 (279.9)	91.4 (152.3)
Carbohydrate % of E	45.6	43.2	53.4	47.7	44.3	40.5	47.4	34.7	44.0^{b1}	42.4	56.4^{ab123}	46.9	45.2^{ab2}	34.6	40.1^{b3}	40.0
Vitamin A(μgRAE)	233.7 (118.0)^{a}	95.7 (48.3)^{c1}	86.8 (43.9)	80.7 (40.8)^{c2}	230.4 (116.4)	128.6 (64.9)	195.0 (98.5)	174.4 (88.1)^{c12}	133.1 (42.6)	119.7 (38.4)	141.7 (45.4)	137.4 (44.0)	131.6 (42.2)	162.8 (52.2)	310.9 (99.7)	163.7 (52.5)
Vitamin B_1(mg)	0.43 (102.4)	0.38 (90.5)	0.34 (81.0)	0.32 (76.2)	0.48 (114.3)	0.40 (95.2)	0.47 (111.9)	0.38 (90.5)	0.53 (94.6)	0.50 (89.3)	0.47 (83.9)^{b}	0.46 (82.1)	0.65 (116.1)	0.46 (82.1)	0.81 (144.6)^{ab}	0.47 (83.9)
Vitamin B_2(mg)	0.36 (85.7)	0.27 (64.3)^{c1}	0.31 (73.8)	0.27 (64.3)^{c2}	0.44 (104.8)	0.34 (81.0)	0.51 (121.4)	0.46 (109.5)^{c12}	0.39 (69.6)^{b}	0.34 (60.7)	0.43 (76.8)	0.40 (71.4)	0.58 (103.6)	0.34 (60.7)	0.80 (142.9)^{ab}	0.47 (83.9)
Vitamin C(mg)	48.1 (171.9)	34.4 (122.9)	50.7 (181.0)^{a}	26.5 (94.6)^{c12}	42.7 (152.4)	41.9 (149.6)^{c1}	76.3 (272.4)^{a}	43.9 (156.8)^{c2}	42.5 (119.8)	48.2 (126.8)	51.7 (136.1)	47.5 (125.0)	42.3 (111.3)	46.3 (121.8)	58.7 (154.3)	54.7 (143.9)
Calcium (mg)	173.0 (48.1)	159.1 (44.2)^{c1}	123.4 (34.3)	107.2 (29.8)^{c12}	247.7 (68.8)^{a}	144.2 (40.1)	239.4 (66.5)	194.7 (54.1)^{c2}	216.6 (54.1)	166.6 (41.7)	162.5 (40.6)	145.4 (36.4)	216.9 (54.2)	205.3 (51.3)	275.4 (68.9)	216.0 (54.0)
Iron (mg)	6.9 (122.3)	6.3 (112.5)	7.0 (124.3)	5.2 (92.9)^{c}	8.3 (148.8)	6.1 (108.9)	10.0 (178.8)	7.4 (132.1)^{c}	7.6 (105.0)	7.6 (105.6)	9.4 (130.6)	8.0 (111.1)	9.8 (136.1)	8.9 (123.6)	14.0 (194.4)	8.8 (122.2)
Zinc (mg)	4.3 (152.9)	4.1 (146.4)	4.9 (174.3)	3.9 (139.3)	5.3 (190.0)	4.2 (150.0)	4.6 (163.6)	4.4 (158.6)	5.7 (167.6)	5.0 (147.1)	7.6 (222.4)	5.6 (165.0)	8.8 (257.6)	5.6 (164.7)	8.4 (248.2)	4.8 (141.2)

Data are presented as median (%). p-value <0.05 is indicative of significance; n - number of schools; (a) there are statistical differences between the same quarters in different regions; (b) there are statistical differences between quarters in urban schools; (c) there are statistical differences between quarters in non-urban schools. The percentage of recommended value = (actual supply/recommended standard value) ×100%.
